# Low serum C3 level, high neutrophil-lymphocyte-ratio, and high platelet-lymphocyte-ratio all predicted poor long-term renal survivals in biopsy-confirmed idiopathic membranous nephropathy

**DOI:** 10.1038/s41598-019-42689-7

**Published:** 2019-04-17

**Authors:** Shang-Feng Tsai, Ming-Ju Wu, Cheng-Hsu Chen

**Affiliations:** 1 Division of Nephrology, Department of Internal Medicine, Taichung Veterans General Hospital, Taichung, Taiwan; 2 Department of Life Science, Tunghai University, Taichung, Taiwan; 3 School of Medicine, National Yang-Ming University, Taipei, Taiwan; 4 School of Medicine, Chung Shan Medical University, Taichung, Taiwan; 5 Department of Lilfe Science, National Chung Hsing University, Taichung, Taiwan

**Keywords:** Immunogenetics, Membranous nephropathy

## Abstract

Idiopathic membranous nephropathy (iMN) is the major cause of end-stage renal disease (ESRD). Recent guidelines suggest limiting immunosuppressants only to high risk patients for ESRD. The present study is aimed at identifying new predictors for the renal outcome of iMN patients. We conducted a retrospective cohort study covering a period from January 2003 to December 2013. We enrolled participants who had received their first renal biopsy at our medical center in Taiwan with the diagnosis of iMN. Clinical, pathological and laboratory data were collected from medical records. Analyses with Mann–Whitney U test was used for continuous variables and Chi-square test for categorical variables. The Kaplan-Meier curve was used for the analyses of patient survival and renal survival. Youden index was used for evaluating the performance of a dichotomous diagnostic test for renal and patient outcomes. Cox proportional hazard regression was used to determine factors affecting renal survival.A total of 99 patients with renal biopsy-confirmed idiopathic iMNs were enrolled. C3 level ≤114 mg/dl predicted patient outcome (p < 0.001) with good predictive power (AUC = 0.736). The univariate analysis showed that risk factors for poor renal outcome were older age (HR = 1.04, p = 0.002), high BUN (HR = 1.03, p < 0.001), poor baseline renal function (HR = 1.30 and p < 0.001 for higher serum creatinine; HR = 0.97 and p < 0.001 for higher eGFR; HR = 1.06 and p < 0.001 for urine PCR), C3 ≤ 93.4 mg/dl (HR = 2.15, p = 0.017), NLR > 3.34 (HR = 3.30, p < 0.001) and PLR > 14.48 (HR = 2.54, p = 0.003). Stage of iMN did not fully account for the risk of ESRD. This is the first evidence that serum levels of C3 ≤ 93.4 mg/dl predicted poor renal outcomes with good predictive power. Easily obtained markers, NLR > 3.34 also predicted poor renal outcomes.

## Introduction

Idiopathic membranous nephropathy (iMN) is the major cause of nephrotic syndrome in adults^1^. Its common pathological finding is the presence of subepithelial deposition over glomerular basement membrane (GBM). The outcome is heterogeneous, spreading from a self-limited status to an end-stage renal disease (ESRD). In addition, the mainstream treatment for iMN is immunosuppressants. However, there is no consensus among medical professionals regarding the timing, types and doses of immunosuppressants. Recently, published in Kidney Disease Improving Global Outcomes (KDIGO) have suggested alkylating agents to be used only in patients with the highest risk for kidney failure in order to avoid unnecessary complications, like infections^2^. Therefore, the identification of the risk factors for renal outcome of iMN is needed.

Toronto Risk Score was proposed in 1997 to predict the renal outcome in patients of iMN^3^. It is based on three parameters: (a) time-averaged proteinuria (highest sustained 6-month period of proteinuria), (b) creatinine clearance (CCr) at diagnosis, and (c) the slope of CCr over 6 months. There is however no long-term follow-up study published for this model. In addition, no validation of this model has been made to include other other non-western races. Increasingly more markers (like including urinaryβ2-microglobulin orα1-microglobulin^4,5^, and anti- phospholipase A2 receptor antibody (anti-PLA2R)^6^ have been developed to strengthen the predictive power. Furthermore, the knowledge of molecular pathogenesis of iMN is actually rare. Recently, differentially expressed microRNAs and their downstream network may be involved in iMN pathogenesis and could be considered as potential diagnostic biomarkers of MGN^7^. Until now, none of the above approaches is perfect, and the outcome of iMN remains poorly predicted. Therefore, other factors responsible for predicting the outcome of iMN needed to be discovered. Here, we conducted a long-term retrospective study on such patients and analyzed the risk factors for their renal outcomes.

## Materials and Methods

### Study population

We conducted a retrospective cohort study covering the period from January 2003 to December 2013. We enrolled participants of ages >20 years and who had received a first renal biopsy for their diagnoses of iMN in our medical center. Graft renal biopsies were excluded. In this medical center, we had the largest population of renal biopsies (>8000 times over 30 years) in Taiwan. We collected patients with the diagnosis of iMN, instead of secondary MN. All recruited patients also did not have other autoimmune disease and infection disease. This study was approved by Ethics Committee of Taichung Veterans General Hospital, IRB number: CE15125B. All methods were carried out in accordance with relevant guidelines and regulations and informed consent was obtained from all subjects.

### Data Collection and outcome assessment

All data were obtained through our reviewing of medical records. At the time of renal biopsy, baseline data were collected. They included the following: gender, age, body height (cm), body weight (kg), and systolic or diastolic blood pressure (SBP and DBP). From blood samples the following data were also collected: blood urea nitrogen (BUN) (mg/dl), serum creatinine (mg/dl), estimated glomerular filtration rate (eGFR from MDRD equation^8^) (ml/min.1.732 m^2^), bloodwhite blood cell (WBC) (/cumm), blood red blood cell (RBC) (/cumm), hemoglobin (g/dl), neutral and lymphocyte ratio (%), platelet count (/cumm), uric acid (mg/dl), sodium (meq/L), potassium (meq/l), calcium (mg/dl), phosphaste (mg/dl), magnesium (mg/dl), albumin (g/dl), total protein (g/dl), glutamate oxaloacetate transaminase (GOT) (U/L), glutamate-pyruvate transaminase (GPT) (U/L), total cholesterol (mg/dl), triglyceride (mg/dl), low-density lipoprotein (LDL) (mg/dl), high-density lipoprotein (HDL) (mg/dl), fasting and postprandial blood sugar (mg/dl), glycated hemoglobin (%). We calculated NLR as the ratio of neutrophil to lymphocyte counts, and PLT as the platelet to lymphocele counts. Markers for chronic infection or inflammation are as follows: hepatitis B status, hepatitis C status, Anti-Nuclear Ab (ANA), anti-double stranded DNA (anti-dsDNA), Anti-neutrophil cytoplasmic antibodies (ANCAs), proteinase 3 (PR3) and myeloperoxidase (MPO). Urinary samples were tested for spot urine protein (mg/dl), spot urine creatinine (mg/dl), spot urine albumin (mg/dl) and 24-hour proteinuria (g/day).

All pathological samples were reviewed by the same pathologist. Participants had the diagnosis of iMN based on stages of iMN (Ehrenreich- Churg)^9^. The study endpoints were patient death and renal death (ESRD). In the latter case, dialysis or kidney transplantation was performed according to our management guidelines.

### Statistical methods

For continuous variables, data were expressed as mean ± SD, and for categorical variables, as numbers (percentages). Mann–Whitney U test was used to analyze the continuous variables and *Chi*-square test for the categorical variables. Kaplan-Meier curve was used to represent patient survival and renal survival. Youden index was used to evaluate the performance of a dichotomous diagnostic test for renal and patient outcomes. Cox proportional hazard regression was used to determine factors affecting renal survivals (univariate and multivariate cox models). Values of p < 0.05 was considered statistically significant. All statistical procedures were performed using the SPSS statistical software package, version 17.0 (Chicago, IL).

## Results

### Demographic data segregated according to stages of iMN

Initially, 187 times of iMN confirmed by renal biopsy patients were screened. Three of them were excluded due to graft kidney biopsy. We further excluded within the remaining 184 native kidney biopsies, the 2^nd^ or 3^rd^ renal biopsies, as well as secondary iMN (excluding autoimmune diseases, infections, medications, and malignancies). Finally, 99 patients with renal biopsy-confirmed idiopathic iMNs were analyzed in this study. The whole duration of follow-up was 11 years (January 2003 to December 2013). The mean duration of follow-up was 6.2 ± 3.2 years. Totally, 79 patients reached endpoint (20 loss of followed or dead). Based on stages of iMN (Ehrenreich- Churg)^9^, we separated 99 participants into two subgroups: subgroup 1 (stage 1–2)^10^ and subgroup 2 (stage 3–4)^10^, as shown in Table 1. As for baseline clinicopathological and laboratory data, recorded at the time of iMN diagnosis, participants were aged around 57.7 year old, and more patients were in the stage 2-chronic kidney disease (eGFR = 75.01 ml/min.1.732 m^2^). Metabolic syndrome was less frequent: i.e. 132.0 mmHg of SBP, 171.18 mg/dl of LDL, and 5.90% of HbA1c. Their proteinuria was severe, 7.99 g/day of daily urine protein. The participants in the more severe stages of iMN had lower percentages of neutrophils (64.62 ± 11.41 vs. 60.24 ± 8.96, p = 0.031) and lower levels of IgM (136.83 ± 91.57 vs. 98.68 ± 55.37, p = 0.011). Patients in stage 3–4 iMN had higher serum creatinine levels (1.10 ± 0.73 vs. 2.05 ± 2.30) but the difference with stage 1–2 was not significant (p = 0.086).

**Table 1:** Demographic characteristics of membranous nephropathy

	Total (n = 99) N %	Stage 1–2 (n = 48) N %	Stage3–4 (n = 51) N %	*p* value
Sex: female	46 (46.5%)	27 (56.3%)	19 (37.3%)	0.091
Age (years old)	57.75 ± 16.57	58.02 ± 15.29	57.49 ± 17.84	0.763
SBP (mmHg)	131.95 ± 18.42	129.65 ± 17.68	134.12 ± 19.00	0.441
DBP (mmHg)	79.43 ± 12.14	77.58 ± 12.07	81.18 ± 12.08	0.176
Body height (cm)	162.09 ± 8.10	162.76 ± 7.04	161.47 ± 9.05	0.524
Body weight (kg)	64.60 ± 12.14	63.29 ± 12.15	65.81 ± 12.13	0.191
BUN (mg/dl)	23.42 ± 19.58	21.08 ± 21.12	25.63 ± 17.95	0.093
Creatinine (mg/dl)	1.59 ± 1.79	1.10 ± 0.73	2.05 ± 2.30	0.086
eGFR (ml/min.1.732 m^2^)	75.01 ± 47.27	81.44 ± 43.99	68.59 ± 49.96	0.179
Blood WBC (/cumm)	7023.5 ± 3151.4	7526.5 ± 3800.5	6550.1 ± 2327.5	0.192
Blood RBC (/cumm)	3.86 ± 0.78	3.92 ± 0.71	3.80 ± 0.85	0.524
Hemoglobin (g/dl)	12.08 ± 2.18	12.50 ± 1.95	11.68 ± 2.33	0.068
Neutrophil (%)	62.34 ± 10.39	64.62 ± 11.41	60.24 ± 8.96	0.031
Lymphocyte (%)	27.14 ± 9.35	26.07 ± 9.82	28.15 ± 8.86	0.217
Platelet (*10^3^/cumm)	255.22 ± 86.08	261.02 ± 87.91	249.76 ± 84.83	0.531
Uric acid (mg/dl)	6.76 ± 1.91	6.55 ± 1.84	6.97 ± 1.98	0.412
Na (meq/L)	140.61 ± 3.54	140.17 ± 3.99	141.02 ± 3.04	0.328
K (meq/L)	4.19 ± 0.59	4.15 ± 0.54	4.23 ± 0.64	0.474
Ca (mg/dl)	7.82 ± 0.99	7.77 ± 0.98	7.87 ± 1.00	0.506
P (mg/dl)	3.82 ± 0.74	3.78 ± 0.66	3.86 ± 0.83	0.578
Mg (mg/dl)	2.23 ± 0.36	2.24 ± 0.26	2.23 ± 0.43	0.500
Albumin (g/dl)	2.51 ± 0.70	2.38 ± 0.60	2.64 ± 0.76	0.096
Total protein (g/dl)	5.30 ± 1.07	5.15 ± 0.94	5.44 ± 1.18	0.246
GOT (U/L)	26.04 ± 14.13	29.15 ± 17.74	23.12 ± 8.84	0.073
GPT (U/L)	22.32 ± 13.18	25.19 ± 15.53	19.67 ± 10.00	0.085
Total cholesterol (mg/dl)	273.69 ± 104.78	284.48 ± 108.85	262.67 ± 100.46	0.391
Triglyceride (mg/dl)	183.86 ± 109.17	191.07 ± 116.11	176.31 ± 102.25	0.487
LDL (mg/dl)	171.18 ± 89.52	182.33 ± 95.79	158.68 ± 81.28	0.288
HDL (mg/dl)	59.97 ± 22.32	62.17 ± 25.79	57.84 ± 18.45	0.466
Fasting glucose (mg/dl)	109.06 ± 40.73	111.29 ± 41.16	106.61 ± 40.66	0.055
Postprandial glucose (mg/dl)	172.93 ± 140.06	150.40 ± 65.65	218.00 ± 234.85	0.951
HbA1c (%)	5.90 ± 0.84	5.92 ± 0.73	5.87 ± 1.00	0.286
IgG (mg/dl)	856.61 ± 513.20	764.64 ± 410.96	948.57 ± 588.48	0.092
IgA (mg/dl)	283.82 ± 116.15	292.38 ± 104.19	275.26 ± 127.57	0.271
IgM (mg/dl)	117.96 ± 77.84	136.83 ± 91.57	98.68 ± 55.37	0.011
IgE (mg/dl)	214.68 ± 349.68	276.88 ± 413.99	156.93 ± 280.89	0.109
C3 (mg/dl)	116.59 ± 29.30	121.00 ± 33.75	112.09 ± 23.45	0.303
C4 (mg/dl)	32.99 ± 11.75	33.60 ± 13.15	32.37 ± 10.27	0.931
HBsAg positive	10 (10.5%)	6 (12.5%)	4 (8.5%)	0.313
Anti-HCV positive	2 (2.1%)	1 (2.1%)	1 (2.1%)	0.552
ANA positive	22 (22.9%)	10 (20.8%)	12 (25.0%)	0.816
dsDNA WHO	22.08 ± 16.72	25.13 ± 18.49	18.07 ± 13.42	0.193
ANCA positive	4 (7.5%)	2 (6.7%)	2 (8.7%)	1.000
MPO (IU/ml)	14.96 ± 27.70	5.14 ± 5.50	39.50 ± 52.89	0.434
PR3 (IU/ml)	21.72 ± 36.54	25.30 ± 39.66	—	—
Rapid plasma reagin positive	3 (9.7%)	1 (6.3%)	2 (13.3%)	0.600
Urine albumin (mg/dl)	334.49 ± 628.03	382.85 ± 714.67	275.39 ± 539.59	0.676
24-hour proteinuria (g/day)	7.99 ± 7.87	8.05 ± 6.36	7.94 ± 9.19	0.527
Urine creatinine (mg/dl)	94.89 ± 66.03	103.61 ± 72.32	86.16 ± 58.54	0.247
Urine PCR (mg/mg)	6.69 ± 6.70	7.45 ± 7.70	5.94 ± 5.54	0.302
Urine ACR (mg/g)	2791.9 ± 4542.3	2198.7 ± 3147.4	3516.8 ± 5961.9	0.676

Chi-square test. Mann-Whitney U test. ^*^p < 0.05, ^**^p < 0.01.

### Patient outcomes

**Figure 1:**
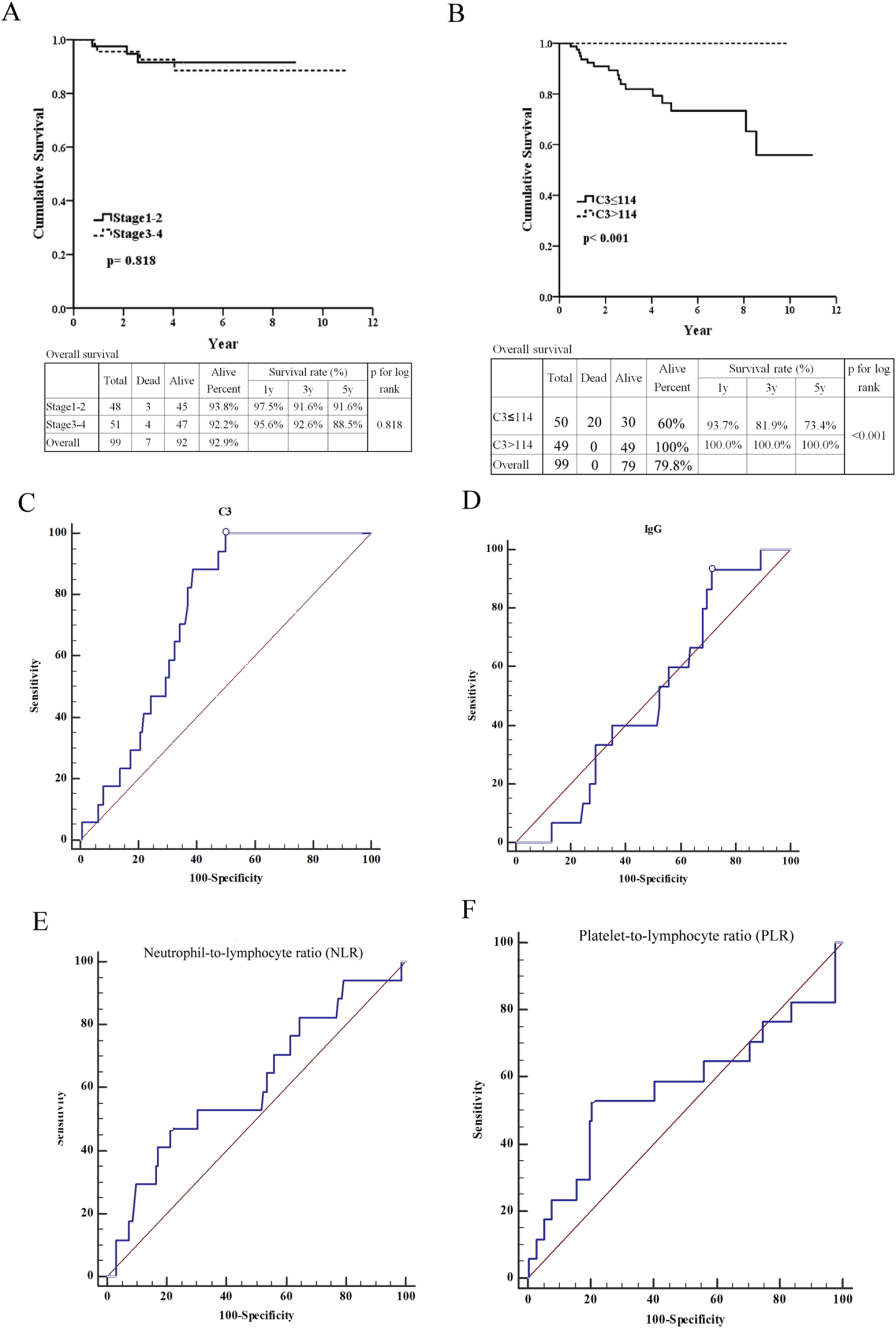
Predictions of patient survival of membranous nephropathy. (**A**) Stages of membranous nephropathy. (**B**) Serum C3 ≤ 114 mg/dl predicts poor patient outcoms (p < 0.001). (**C**) Serum C3 ≤ 114 mg/dl predicts poor patient outcoms with good diagnostic power (AUC = 0.736, sensitivity = 100%, specificity = 50%). (**D**) IgG predicted patient survival with poor diagnostic power (AUC = 0.501, sensitivity = 93.33%, specificity = 28.48%). (**E**) Neutrophil-to-lymphocyte ratio predicts patient survival with poor diagnostic power (AUC = 0.612, sensitivity = 47.06%, specificity = 78.66%). (**F**) Platelet-to-lymphocyte ratio predicts patient survival with poor diagnostic power (AUC = 0.570, sensitivity = 52.94%, specificity = 79.52%).

**Figure 2:**
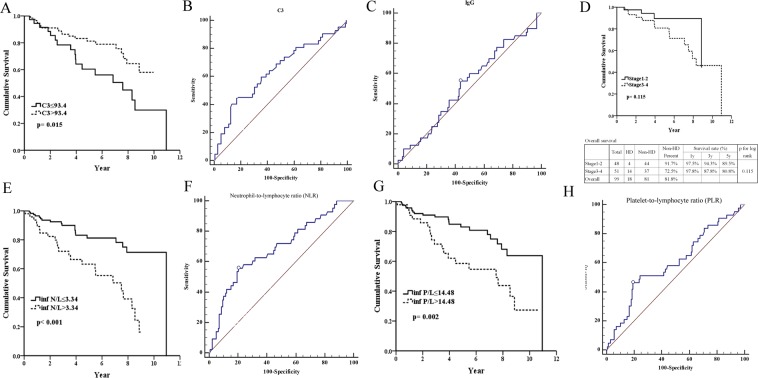
Predicting renal survival of membranous nephropathy. (**A**) Serum C3 ≤ 93.4 mg/dl predicts poor renal survival (p = 0.015). (**B**) Serum C3 ≤ 93.4 mg/dl predicts poor renal survival with good diagnostic power. (AUC = 0.643, sensitivity = 45.24%, specificity = 82.96%). (**C**) Serum IgG > 730 mg/dl predicts renal survival with poor diagnostic power (AUC = 0.526, sensitivity = 55%, specificity = 56.35%). (**D**) Stages of membranous nephropahy do not predict renal survival. (**2E**) Neutrophil-to-lymphocyte ratio >3.34 predicts poor renal survival (p < 0.001). (**F**) Neutrophil-to-lymphocyte ratio >3.34 predicts poor renal survival with good diagnostic power (AUC = 0.684, sensitivity = 55.81%, specificity = 79.71%). (**G**) Platelet-to-lymphocyte ratio >14.48 predicts poor renal survival (p = 0.002). (**H**) Platelet-to-lymphocyte ratio >14.48 predicts poor renal survival with fair diagnostic power. (AUC = 0.60, sensitivity = 46.51%, specificity = 80.71%).

As shown in Fig. 1A, we found according to the pathological stages of iMN, the 1-year, 3-year and 5- year patient survivals as follow: 97.5%, 91.6%, and 91.6% for stage 1–2; 95.6%, 92.6 and 88.5% for stage 3–4. Five-year after the diagnosis of iMN, patients of stage 3–4 appeared to have worse survivals, but no statistical significant difference was found with those of stage 1–2 (p = 0.818). No statistical significant difference was found in patient survivals across the stages of iMN. The predictive powers of C3, IgG, neutral-to-lymphocyte ratio (NLR), and platelet-to-lymphocyte ratio (PLR) were weak (AUC = 0.568, 0.648, 0.684, and 0.673, respectively) (Fig. 2). The C3 ≤ 114 mg/dl also predicted poor patient outcomes (p < 0.001) (Fig. 1B) with good predictive power (AUC = 0.736, sensitivity = 100%, and specificity = 50%) (Fig. 1C). IgG, NLR, and PLR did not predict patient outcomes (Fig. 1D to F).

### Renal outcomes

Table 2 shows the results of univariate analysis: risk factors for poor renal outcomes were old ages (HR = 1.04, p = 0.002), high BUN (HR = 1.03, p < 0.001), poor baseline renal function (HR = 1.30 and p < 0.001 for higher serum creatinine; HR = 0.97 and p < 0.001 for higher eGFR; HR = 1.06 and p < 0.001 for urine PCR), C3 ≤ 93.4 mg/dl (HR = 2.15, p = 0.017), NLR > 3.34 (HR = 3.30, p < 0.001) and PLR > 14.48 (HR = 2.54, p = 0.003). The multivariate analysis revealed risk factors for poor renal functions being poor baseline renal function (HR = 0.97, p = 0.011 for higher eGFR), and NLR > 3.34 (HR = 2.25, p = 0.042). Youden index for C3 level was ≤93.4 mg/dl with good diagnostic power (AUC = 0.643) (Fig. 2B) in predicting poor renal outcomes (p = 0.015) (Fig. 2A). In addition, Youden index for NLR level was >3.34 with good diagnostic power (AUC = 0.684) (Fig. 2F) in predicting poor renal outcomes (p < 0.001) (Fig. 2E). Youden index for PLR level was >14.48 with good diagnostic power (AUC = 0.600) (Fig. 2H) in predicting poor renal outcomes (p < 0.002) (Fig. 2G). Whereas, IgG level (Fig. 2C) and pathological stages of iMN (Fig. 2D) showed no differences.

**Table 2:** Cox regression models for renal survival via univariate and multivariate analysis.

	Univariate			Multivariable		
	Hazard ratio	95%CI	*P* value	Hazard ratio	95%CI	*P* value
Male	1.29	0.69–2.42	0.428			
Age	1.04	1.01–1.06	0.002	1.02	0.99–1.05	0.301
Blood urea nitrogen	1.03	1.02–1.03	<0.001			
Serum creatinine	1.30	1.19–1.41	<0.001	0.91	0.72–1.14	0.408
eGFR	0.97	0.96–0.98	<0.001	0.97	0.95–0.99	0.011
Uric acid	0.95	0.81–1.12	0.549			
Albumin	0.96	0.61–1.51	0.856			
Total protein	0.77	0.56–1.07	0.116			
LDL	1.00	1.00–1.00	0.427			
Urine PCR	1.06	1.04–1.09	<0.001	1.03	1.00–1.07	0.068
Daily urine protein	1.02	0.99–1.05	0.244			
IgG	1.00	1.00–1.00	0.541			
IgA	1.00	0.99–1.00	0.053			
C3 ≤ 93.4 mg/dl	2.15	1.14–4.02	0.017	0.95	0.46–1.94	0.884
C4	1.02	1.00–1.04	0.084			
NLR > 3.34	3.30	1.78–6.13	<0.001	2.58	1.04–6.42	0.042
PLR > 14.48	2.54	1.38–4.69	0.003	0.92	0.40–2.09	0.837
Stage 3–4/1–2	2.41	0.78–7.43	0.127			

Cox proportional hazard regression. ^*^p < 0.05, ^**^p < 0.01.

## Discussion

That baseline poor renal function (higher BUN, higher creatinine, higher proteinuria, and lower eGFR) were risk factors for poor long term renal function, is a finding of our study in consistence with other studies^3,10^, including Toroto Risk Score with 85–90% of the accuracy^3^. However, we believed more new parameters are needed. Firstly, this score was based on clinical and laboratory variables in Western population of Italy, Finland and Toronto. The application to other races is not clear. Besides, parameters in this model are late markers. This model was developed 20 years ago and the outcome prediction of iMN had not improved since. Thirdly, most clinical skepticism of this model is the lack of long-term studies. Finally, they only collected common parameters for analyzed (background renal function, blood pressure, and ages). Therefore, more and newer biomarkers to predict outcomes of iMN are needed.

The morphologic severity of histologic sample with the diagnosed of iMN is related to long-term renal outcomes^10,11^. Staging of iMN (Ehrenreich-Churg) was reported more than 40 years ago^9^, and it was focused only on the status of GBM. The predictive value for renal outcome has not been discussed except by Marx *et al*. in 1999^10^. The HR for ESRD of stage III/IV vs. I/II was found to be high at 5.3 (p = 0.002). After that, no study has validated the predictive power of such staging system. In our study, we found no statistical significance between different disease stages (HR = 2.41, 0.78–7.46 of 95% CI, p = 0.127). We believe the background histological severity is not reflected only GBM status, but also by glomerular segmental sclerosis and tubulointerstitial injury both of which can affect the long-term renal outcome. According to Chen *et al*.^11^, over a median follow-up of 34 months, segmental sclerosis and tubulointerstitial injury are independent risk factors respectively for 20 and 50% of renal function decline and the progression to ESRD. In brief, GBM status (Ehrenreich-Churg) alone failed to fully predict long-term renal outcome.

This present study is the first of its kind to show that serum C3 ≤ 93.4 mg/dl predicted poor renal outcome (HR = 2.15, p = 0.017) with good predictive power (AUC = 0.643). The complement system is important for the pathogenesis of iMN. Subepithelial immune deposits activate the complement system which starts activation of the C3 component, conversion of C5, and subsequent formation of the C5b-9 complex in the podocyte membrane^12–15^. In 1984, C3 deposits were first detected in half of those patients with idiopathic iMN^16,17^. Furthermore, C3d deposits were also detected in all patients associated with IgG deposits^17^. Patients with glomerular C3 deposits exhibited more severe proteinuria, which supported the association of complement activation with disease severity in human iMN^17^. Recently, using more sensitive immunohistological stainings, C3 deposits can be detected in almost all cases of iMN^18,19^. The greater the deposition of C3, the lower the serum C3 level, which predicted poor long-term renal function. In addition, low C3 level is a risk factor for poor patient survival as found in our study. Patients with poor renal function may tend to receive more immunosuppressants in treatment and consequently wound develop moreimmunosuppressant related complications, such as infections.

Both mean value of NLR > 3.34 (p < 0.01) and PLR > 14.48 (p = 0.002) predicted poor renal outcome. This is the first study to report such findings. We are also the first to demonstrate that NLR levels predicted renal outcome in iMN patients. NLR is a simple and inexpensive laboratory marker of the inflammatory status, including cirrhosis^20^, cardiovascular disease^21^, malignancy^20,22,23^, community-acquired pneumonia^24^, carotid atherosclerosis^25^ and stroke-associated pneumonia^26^. Its normal levels are between 0.78 and 3.53^27^. NLR predicts renal prognosis in lupus nephritis^28^ and granulomatosis with polyangiitis^29^. In addition, NLR is a risk factor for community-acquired acute kidney injury and NLR is associated with poor recovery of renal function^30^. Neutrophilia and lymphocytopenia are both indicative of disease severity. Also, an increased NLR level may be related to a low lymphocyte count. It has been proposed that under physiologic stress, the induced increase in cortisol secretion causes lymphopenia^31^. Similarly, PLR is also considered an inflammatory marker in thrombotic events, inflammatory diseases, and malignancies^32,33^. Recently, PLR has been associated with all-cause of mortality in the elderly with chronic kidney disease^34^, and it is associated with higher C-reactive protein in ESRD patients^35^. Platelets are viewed as immune cells and can interact with other types of immune cells, including endothelial cells, T-cells, neutrophils, and mononuclear phagocytes. These interactions might initiate and exacerbate the inflammation in the arterial wall^36^. Although without definite association, we still recommend routine checks for iMN patients, of such easily obtainable markers (like NLR and PLR), to improve the predictive power of renal outcome,especially NLR. NLR > 3.34 may have higher predictive power than PLR > 14.48. Firstly, both multivariable and univariate Cox regression models showed that NLR is a significant predictor of renal survival. NLR had predictive power only in univariate Cox regression. Besides, the predictive power (AUC) of NLR > 3.34 is higher than PLR > 14.48 (0.684 vs. 0.600)

There are several limitations of this study. Firstly, the subjects were chosen from a single medical center even though the samples of renal biopsies were relatively large in number (>8000 over 30 years). Secondly, there was insufficient data on treatments. The standard treatment for iMN was consistent according to current guidelines in this medical center. Angiotensin-converting Enzyme inhibitors (ACEis) or angiotensin II Receptor Blockers (ARBs) were prescribed to all patients with iMN. This immunosuppressive therapy was given for those experiencing a progressively declining GFR, or refractory and persistent proteinuria above 4 g/day after maximal ACEis or ARBs. Thirdly, we did not measure anti-PLA2R antibody^37^. However, in clinical practice, we did not measure this marker. Therefore, our study reflected results in the real world. Finally, we did not record complications of immunosuppressants, which could cause more infection-related mortality.

## Conclusions

Stages of iMN did not fully explain the risks for ESRD. This is the first study to identify serum levels of C3 ≤ 93.4 mg/dl predicted poor renal outcome with good predictive power. Easily obtained markers, NLR > 3.34 predicted poor renal outcomes.
